# An Adaptive Rate Blocked Compressive Sensing Method for Video

**DOI:** 10.3390/e23081002

**Published:** 2021-07-31

**Authors:** Jianming Wang, Jianhua Chen

**Affiliations:** School of Information Science and Engineering, Yunnan University, Kunming 650500, China; chenjh@ynu.edu.cn

**Keywords:** compressive sensing, statistical parameter estimation, sparsity estimation, adaptive rate sampling, video

## Abstract

An adaptive rate Compressive Sensing (CS) method for video signals is proposed. The Blocked Compressive Sensing (BCS) scheme is adopted in this method. Firstly, each video frame is blocked and measured by the BCS scheme, and then the mean and variance of each image block are estimated by observing the CS measurement results. Using the mean and variance of each image block, the sparsity of the block is estimated and then the block can be classified. Adaptive rate sampling is realized by assigning different sampling rates to different classes. At the same time, in order to make better use of the correlation between video frames, a reference block subtraction method is also designed in this paper, which uses the estimates of the sparsity of image blocks as the basis for the reference block update. All operations of the proposed method only depend on the CS measurement results of image blocks and all calculations are simple. Thus, the proposed method is suitable for implementation in CS sampling devices with limited computational performance. Experiment results show that, compared with the actual values, the sparsity estimates and block classification results of the proposed method are accurate. Compared with the latest adaptive Compressive Video Sensing methods, the reconstructed image quality of the proposed method is better.

## 1. Introduction

Compared with the traditional Compressive Sensing (CS) [1,2,3], the adaptive CS can adapt to the changes of the signal more effectively and achieve more reasonable signal sampling by using an appropriate CS matrix, sparse basis, sparse dictionary or sampling rates, to reduce the overall sampling rates and improve the quality of reconstructed image. In this paper, the “sampling rate” is defined as the ratio of the number of CS measurements to the length of the original signal.

Due to the characteristics of the CS imaging method [4], the original signal can be sampled without digital conversion and storage, thus the complexity of sampling calculation can be greatly reduced, the hardware requirements of the sampling equipment can be simplified, and the sampling rate can be improved. This makes the CS method have unique advantages in fields such as video compression [5,6], distributed coding [7], sensor networks [8], radar imaging [9], medical imaging [10], etc.

According to measurement models, CS methods can be divided into ones using global measurement scheme [11] and some others using block compressive sensing scheme (BCS) [12,13]. In general, global measurement methods can get better performance in the non-adaptive case [14]. However, the size of the measurement matrices in global measurement methods are often very large, so the total number of matrix multiplication in the sampling process will be large, and memory occupation of the measurement matrix is also large. In this paper, the BCS scheme is used to reduce the size of the measurement matrix and reduce the sampling computation. By allocating appropriate sampling rate to different blocks, the disadvantage of the BCS scheme can be overcome, and the performance of the method is greatly improved.

However, it is often difficult to implement adaptive rate sampling in the CS method. In CS applications, the original signal can be regarded as an unknown signal, which makes it difficult to implement adaptive methods. For the sampling device, the information that can be directly obtained is no longer the original signal, but the result of the CS measurement, which is called the “CS domain signal”. In earlier studies, researchers mainly used the CS domain signal to reconstruct the original signal, then used the reconstructed signal to estimate the characteristics of the original signal, and adaptively adjusted the CS matrix [15,16,17], sparse basis [18,19], sparse dictionary [20,21] or sampling rate [22,23]. The advantage of these methods is that it can obtain the characteristics of the original signal with high accuracy, and then make full use of the characteristics of the signal to adjust one or more sampling elements. In addition, the accuracy of these adjustments is also high, which can follow the changes of the original signal well to make corresponding adjustments. However, an important problem of these methods is that the computational complexity of the signal reconstruction is quite high, and it is unlikely to be implemented in the sampling device of CS. If the adaptive adjustment is implemented in the decoder, a special feedback channel is needed, which will also greatly affect the real-time performance of the sampling process.

In order to solve the above problem, some researchers have proposed new research ideas in recent years. They hope that some interesting features can be extracted from the CS domain signal through some simple calculations to guide the sampling device to carry out adaptive sampling, to avoid the dependence on the reconstruction of the original signal.

According to this consideration, researchers have put forward some adaptive CS methods based on CS domain signals in recent years. In [24], researchers proposed a method to estimate the statistical parameters of the original signal by using the CS domain signal, and then estimate the adaptive sampling rate. For image compressive sensing, the covariance of the original signal is estimated by designing a special autoregressive CS matrix, and then an appropriate sampling rate is allocated for each image block. In [25], researchers proposed a method to estimate the motion of objects in the original video depending on the CS domain signal, and then judge the motion speed of objects and realize adaptive rate CS. In [26], researchers proposed a BCS method for video signal, which judges the complexity of the original signal through the change of the CS domain signal in the spatial and temporal dimensions, and adjusts the sampling rate of each image block in real-time to realize adaptive rate CS. In our previous work [27], an adaptive rate CS method for surveillance video is proposed, which uses the change of innovation energy to estimate the complexity of innovation, and then realizes adaptive rate CS. The signal foreground is obtained by the background subtraction method [28]. The total energy of the foreground signal is estimated by using the CS domain signal, and then the number of large value points of the original signal are estimated.

These methods avoid the dependence on signal reconstruction, but there are still some problems in these methods, such as the dependence on some specially designed sampling matrix, inaccurate rate estimation, only suitable for simple surveillance video signals etc. Under the condition that the computing capacity of sampling equipment is strictly limited, the adaptive method of adjusting the sampling rate based on the CS domain signal is more difficult than the method based on the reconstructed signal, and the number of related research works is relatively small. In [24,26], researchers have mentioned that in the field of image CS and video CS, few researchers have reported the relevant research before them.

In this paper, a new adaptive rate CS method based on the BCS scheme is proposed which only uses the CS domain signal and some simple operations to realize adaptive rate sampling and achieves better reconstruction performance. The basic idea is as follows: the original signal is CS measured firstly, the number of measurements equals the length of the original signal, this kind of measurement is called “the full-speed measurement” in this paper. Then, the full-speed measurement result (the CS domain signal) is used to estimate the statistic characteristics of the original signal. According to the estimation of the statistic characteristics, the sparsity of the original signal is estimated in the wavelet transform domain and then the original signal can be classified. Different CS sampling rates are allocated for different signal classes. Finally, according to the allocated sampling rate, the redundant part of the full-speed measurement result is discarded and only the imperative samples are transmitted for reconstruction. Thus, adaptive rate CS can be implemented.

In order to make full use of the statistic characteristics of the wavelet transform coefficients, a new CS measurement scheme is designed, which can estimate the statistic characteristics of different subbands more accurately.

In order to make full use of the inter frame correlation of video signals, a dynamic reference block update and subtraction method is designed. Compared with the measurement method using only intra frame information, the proposed method may occupy more memory, however it can make better use of the inter frame correlation to reduce the total sampling rate. Meanwhile, in the proposed method, reference blocks are automatically updated, the total number of reference blocks stored in memory is equal to the number of blocks in an image frame, so the memory space occupied by the reference blocks is fixed and acceptable.

The rest parts of the paper are organized as follows: in the second section, we briefly introduce the BCS method. In the third section, we introduce the proposed adaptive rate compressive video sensing (CVS) method. In the fourth section, the experiment results and corresponding analysis of the results are given. The final section summarizes the whole work.

## 2. BCS

In CVS applications, in order to reduce the memory occupation of the measurement matrix and the amount of sampling computation, it is necessary to reduce the size of the signal. BCS is a common method. For a two-dimensional video frame $v_{t}\in R^{h\times c}$ with h rows and c columns at time t, it can be decomposed into K non-overlapping image blocks. The size of each image block is B rows and B columns, and the i-th (i∈1,2,⋯,K) image block is recorded as $v_{t}^{i}\in R^{B\times B}$. Theoretically, the value of B can be any positive integer, and in image and video processing, the value of B is often the power of 2, such as 8 or 16 are commonly used. In this paper, we take B=8.

In the process of the CS measurement, we first convert the image block $v_{t}^{i}$ into a vector $x_{t}^{i}\in R^{B^{2}}$, and then we can use the measurement matrix $\Phi_{1}\in R^{M_{1}\times B^{2}}$ and an appropriate sparse basis $\Psi_{1}\in R^{B^{2}\times B^{2}}$ to measure it, the measurement result $y_{1}{}_{t}^{i}$ can be obtained as

(1) $$y_{1}{}_{t}^{i}=\Phi_{1}\Psi_{1}x_{t}^{i}=A_{1}x_{t}^{i}=\Phi_{1}c_{t}^{i},$$

where $A_{1}=\Phi_{1}\Psi_{1}$ and $c_{t}^{i}=\Psi_{1}x_{t}^{i}$, $c_{t}^{i}$ is the sparse representation coefficients of $x_{t}^{i}$ on the sparse basis $\Psi_{1}$. $M_{1}\leq B^{2}$, this means that the signal is compressed into a low dimensional space.

## 3. Adaptive Rate Compressive Video Sensing

Because each frame in the actual video has different content, for blocks in a frame, it is possible that they have quite different characteristics, and the sparsities of different blocks are also completely different. In order to adapt to the changes in different blocks, a new adaptive rate BCS method is proposed to estimate the appropriate sampling rate for each block. At the same time, using the estimate of the sparsity of a block, a new method using inter frame correlation is also proposed.

### 3.1. Statistic Parameter Estimation Based on Restricted Isometry Property

For a signal vector $x_{t}^{i}$, assume that it is unknown to the sampling device and only its CS measurement result is obtained. In order to estimate an appropriate sampling rate for the signal, the sparsity of the sparse coefficient $c_{t}^{i}$ needs to be estimated without reconstructing $x_{t}^{i}$. In this paper, we use the restricted isometry property (RIP) [29] to estimate the mean and variance of $c_{t}^{i}$, and then to estimate the sparsity of $c_{t}^{i}$.

For a CS measurement matrix $\Phi \in R^{B^{2}\times B^{2}}$ and an appropriate sparse basis $\Psi \in R^{B^{2}\times B^{2}}$, we have A=ΦΨ. If A satisfies the RIP, for a Restricted Isometry Constant (RIC) $\delta_{s}\in \left(0,\;1\right)$, there is:

(2) $$1-\delta_{s}\leq \frac{\|Ax_{t}^{i}{\|}_{2}^{2}}{\|x_{t}^{i}{\|}_{2}^{2}}\leq 1+\delta_{s}.$$

In practice, we can approximate it as

(3) $$\|x_{t}^{i}{\|}_{2}^{2}≅\|Ax_{t}^{i}{\|}_{2}^{2}.$$

Set a vector $d_{1}\in R^{B^{2}}$ with $d_{1}=n_{1}D$, where $D={\left[1,1,\ldots ,1\right]}^{T}$ and $n_{1}$ is a constant, and we have

(4) $${\left\|(x_{t}^{i}-d_{1})\right\|}_{2}^{2}≅\|A{\left(x_{t}^{i}-d_{1})\right\|}_{2}^{2}.$$

From Equations (3) and (4), we have

(5) $${\left\|(x_{t}^{i}-d_{1})\right\|}_{2}^{2}-\|x_{t}^{i}{\|}_{2}^{2}≅\|Ax_{t}^{i}-Ad_{1}{\|}_{2}^{2}-\|Ax_{t}^{i}{\|}_{2}^{2}.$$

Since $x_{t}^{i}={\left[x_{t}^{i}{}_{1},x_{t}^{i}{}_{2},\ldots ,x_{t}^{i}{}_{B^{2}}\right]}^{T}$, Equation (5) can be written as:

(6) $$-2n_{1}(x_{t}^{i}{}_{1}+x_{t}^{i}{}_{2}+\cdots +x_{t}^{i}{}_{B^{2}})+B^{2}n_{1}{}^{2}≅\|Ax_{t}^{i}-Ad_{1}{\|}_{2}^{2}-\|Ax_{t}^{i}{\|}_{2}^{2}.$$

If all elements in vector $x_{t}^{i}$ are independent realizations of a random variable $X_{t}^{i}$, denote the expectation of $X_{t}^{i}$ as $E\left(X_{t}^{i}\right)$, it is easy to get the estimate of the expectation $E^{*}\left(X_{t}^{i}\right)$ with

(7) $$E\left(X_{t}^{i}\right)=\frac{\left(x_{t}^{i}{}_{1}+x_{t}^{i}{}_{2}+\cdots +x_{t}^{i}{}_{B^{2}}\right)}{B^{2}}≅\frac{\|Ax_{t}^{i}-Ad_{1}{\|}_{2}^{2}-\|Ax_{t}^{i}{\|}_{2}^{2}-B^{2}n_{1}{}^{2}}{-2n_{1}B^{2}}=E^{*}\left(X_{t}^{i}\right).$$

Set up another vector $d_{2}\in R^{B^{2}}$, $d_{2}=E^{*}\left(X_{t}^{i}\right)D$, Then the estimate of the variance of $X_{t}^{i}$ can be recorded as

(8) $$D^{*}\left(X_{t}^{i}\right)=\|x_{t}^{i}-d_{2}{\|}_{2}^{2}/B^{2}.$$

For

(9) $${\left\|(x_{t}^{i}-d_{2})\right\|}_{2}^{2}≅\|A{\left(x_{t}^{i}-d_{2})\right\|}_{2}^{2},$$

we can take

(10) $$D^{*}\left(X_{t}^{i}\right)≅\frac{\|Ax_{t}^{i}-Ad_{2}{\|}_{2}^{2}}{B^{2}}.$$

These allow us to estimate the characteristics of $x_{t}^{i}$ using only the CS domain signal.

### 3.2. Statistic Characteristics Estimation for Wavelet Subbands

The signal $x_{t}^{i}$ is often not sparse, but it can be represented by a sparse coefficient vector $c_{t}^{i}$ under a sparse base. If all elements in vector $c_{t}^{i}$ are independent realizations of a random variable $C_{t}^{i}$, similar to Equations (7) and (10), the mean and variance of $C_{t}^{i}$ can be estimated using

(11) $$E^{*}\left(C_{t}^{i}\right)=\frac{\|\Phi c_{t}^{i}-\Phi d_{1}{\|}_{2}^{2}-\|\Phi c_{t}^{i}{\|}_{2}^{2}-B^{2}n_{1}{}^{2}}{-2n_{1}B^{2}}\;$$

and

(12) $$D^{*}\left(C_{t}^{i}\right)≅\frac{\|\Phi c_{t}^{i}-\Phi d_{3}{\|}_{2}^{2}}{B^{2}}\;$$

where $d_{3}\in R^{B^{2}}$, and $d_{3}=E^{*}\left(C_{t}^{i}\right)D$.

In this paper, the wavelet basis is used to sparsely represent the original signal. We have noticed that for natural images and videos, there is some specific feature in their wavelet coefficient vector $c_{t}^{i}$, which mainly shows the energy concentration property. Based on the characteristics of the wavelet transform, $c_{t}^{i}$ can be divided into several subbands according to the number of layers of the wavelet transform. The feature of energy concentration is that the main energy of the signal is concentrated in the lower frequency subbands, and the small amount of energy is left in the higher frequency subbands. The absolute values of the coefficients in lower frequency subbands tend to be larger, while the absolute values of the coefficients in the higher frequency subbands tend to be smaller. At the same time, within a subband, the absolute values of nonzero coefficients tend to be close, while the absolute values of nonzero coefficients between different subbands differ greatly.

Due to the above characteristics of the wavelet coefficients, estimating the sparsity of $c_{t}^{i}$ directly using the mean and variance of all coefficients in $c_{t}^{i}$ often leads to results that are not accurate enough. However, if the mean and variance of the coefficients in each subband can be obtained, the sparsity of each subband can be estimated separately, and then the sparsity of the entire wavelet transform coefficient vector $c_{t}^{i}$ can be more accurately estimated.

In order to obtain the mean and variance for each subband, a new CS measurement process is proposed. Consistent with previous assumptions, there is no need to know the digital conversion results of the original signal and the corresponding wavelet transformation coefficients during the measurement process.

For an L-layer wavelet transform, denote the wavelet transform matrix as $\Psi_{w}\in R^{B^{2}\times B^{2}}$, the wavelet transform coefficient vector is

(13) $$c_{t}^{i}=\Psi_{w}x_{t}^{i}.$$

According to the rule of the wavelet transform, the coefficients can be divided into L+1 subbands. Denote one of the coefficients subbands as $c_{t}^{il}\left(l=0,\;1,\cdots ,\;L\right)$,

(14) $$c_{t}^{il}=c_{t}^{i}{}_{(b_{l}+1):b_{l+1}},$$

where $c_{t}^{i0}$ is the lowest frequency subband and $c_{t}^{iL}$ is the highest frequency subband, $c_{t}^{i}{}_{(b_{l}+1):b_{l+1}}$ represents a new vector composed by elements from the $\left(b_{l}+1\right)$-th element to the $b_{l+1}$-th element in $c_{t}^{i}$. And $b_{j}$ is given by

(15) $$b_{j}=\left\{\begin{array}{c} \;\;0,\;\;\;\;j=0\\ \frac{B^{2}}{2^{\left(L-j+1\right)}},\;L+1\geq j\geq 1 \end{array}\right.$$

Set

(16) $$\Psi_{w}^{l}=\Psi_{w}{}_{(b_{l}+1):b_{l+1}},$$

where $\Psi_{w}{}_{(b_{l}+1):b_{l+1}}$ is a new matrix composed by the vectors from the $\left(b_{l}+1\right)$-th row to the $b_{l+1}$-th row in $\Psi_{w}$. Then we have

(17) $$c_{t}^{il}=\Psi_{w}^{l}x_{t}^{i}$$

If there is a random CS matrix $\Phi_{r}\in R^{B^{2}\times B^{2}}$, set a submatrix $\Phi_{r}^{l}\in R^{B^{2}\times (b_{l+1}-b_{l})}$ which is composed by the vectors of column $\left(b_{l}+1\right)$ to $b_{l+1}$ in $\Phi_{r}$,

(18) $$\Phi_{r}^{l}={\left(\Phi_{r}{{}^{T}}_{(b_{l}+1):b_{l+1}}\right)}^{T}\;$$

where $\Phi_{r}{}^{T}$ is the transpose of $\Phi_{r}$.

Then $c_{t}^{il}$ can be measured by $\Phi_{r}^{l}$

(19) $$y_{t}^{il}=\Phi_{r}^{l}c_{t}^{il}=\Phi_{r}^{l}\Psi_{w}^{l}x_{t}^{i}=A_{\mathrm{rw}}^{l}x_{t}^{i}$$

where $A_{\mathrm{rw}}^{l}=\Phi_{r}^{l}\Psi_{w}^{l}$. The CS measurement results of a certain wavelet coefficient subband can be obtained directly from the CS matrix and the wavelet transform matrix. We can know from the matrix multiplication rules that,

(20) $$y_{t}^{i}=\overset{L}{\underset{l=0}{\sum }}y_{t}^{il}=\Phi_{r}c_{t}^{i}=\Phi_{r}\Psi_{w}x_{t}^{i}$$

which means the CS measurement result of $c_{t}^{i}$ can be obtained from the CS measurement results of $c_{t}^{il}$.

If all elements in vector $c_{t}^{il}$ are independent realizations of a random variable $C_{t}^{il}$, Similar to Equations (11) and (12), the mean and variance of $C_{t}^{il}$ can be obtained as follows,

(21) $$E^{*}\left(C_{t}^{il}\right)=\frac{\|y_{t}^{il}-\Phi_{r}^{l}d_{1}^{l}{\|}_{2}^{2}-\|y_{t}^{il}{\|}_{2}^{2}-(b_{l+1}-b_{l})n_{1}{}^{2}}{-2n_{1}(b_{l+1}-b_{l})}$$

where $d_{1}^{l}=n_{1}D^{l}$, $D^{l}\in R^{\left(b_{l+1}-b_{l}\right)}$, $D^{l}={\left[1,\;1,\ldots ,\;1\right]}^{T}$, and

(22) $$D^{*}\left(C_{t}^{il}\right)≅\frac{\|y_{t}^{il}-\Phi_{r}^{l}d_{4}^{l}{\|}_{2}^{2}}{b_{l+1}-b_{l}}\;$$

where $d_{4}^{l}=E^{*}\left(C_{t}^{il}\right)D^{l}$.

### 3.3. Sparsity Estimation

In the process of the CS measurement, a threshold τ is often set, if the value of the measured signal sample is greater than τ, it is considered as a large value, otherwise it is considered as a small value. The value of τ determines how much energy in the original signal is considered as “noise”. If the value of τ is too small, many image blocks will be mistakenly considered as not sparse, resulting in unreasonable increase of sampling rate. If the value is too large, many pixels will be considered as small value affected by noise, which will eventually affect the quality of the reconstructed image. Therefore, the value of τ should be a small value that matches the reconstruction algorithm. In this paper, considering that the original signals are 256 level gray images, the value of τ is set to 8.

The number of large values in the measured signal determines the sparsity of the signal, which in turn, determines the number of measurements.

In the proposed method, we assume that coefficient values in $c_{t}^{il}$ obey a certain distribution. For the wavelet coefficients, it is generally considered that Bessel K form densities (BKF) [30] or generalized gaussian density (GGD) [31] can better describe their distributions. However, it is difficult to get better estimates of the parameters which are necessary in the BKF and GGD distributions when only the CS domain signals are known, thus, we use the normal distribution to describe the coefficient distribution in $c_{t}^{il}$. Since the actual distribution of elements in $c_{t}^{il}$ cannot be optimally approximated with a normal distribution, there will also be some error in the estimated result of the sparsity. However, in this paper, the estimated result of the sparsity is used to classify the image block instead of accurately solving the sampling rate, so approximating the real distribution using the normal distribution is still an effective method.

For an image block, its wavelet coefficients contain L+1 subbands, one of the subband is $c_{t}^{il}$, we assume that coefficient values in $c_{t}^{il}$ are normal distributed, $c_{t}^{il}\sim N\left(E^{*}\left(C_{t}^{il}\right),D^{*}\left(C_{t}^{il}\right)\right)$. The probability of an element in $c_{t}^{il}$ taking a large value is

(23) $$P(c_{t}^{il})=1-\int_{-\tau }^{\tau }N\left(E^{*}\left(C_{t}^{il}\right),D^{*}\left(C_{t}^{il}\right)\right)dx$$

Denote the estimate of the number of large points in $c_{t}^{i}$ by

(24) $$\mathrm{LPN}{\left(c_{t}^{i}\right)}^{*}=\sum_{l=0}^{L}(b_{l+1}-b_{l})P(c_{t}^{il})\;$$

In this paper, we classify blocks into four categories by $\mathrm{LPN}{\left(c_{t}^{i}\right)}^{*}$, denoted C0, C1, C2, and C3. For three sparsity thresholds ${\mathrm{st}}_{1}$, ${\mathrm{st}}_{2}$ and ${\mathrm{st}}_{3}$, where $0<{\mathrm{st}}_{1}<{\mathrm{st}}_{2}<{\mathrm{st}}_{3}<B^{2}$. If $0<\mathrm{LPN}{\left(c_{t}^{i}\right)}^{*}\leq {\mathrm{st}}_{1}$, $c_{t}^{i}$ can be classified into the C0 class, if ${\mathrm{st}}_{1}<\mathrm{LPN}{\left(c_{t}^{i}\right)}^{*}\leq {\mathrm{st}}_{2}$, $c_{t}^{i}$ can be classified into the C1 class, if ${\mathrm{st}}_{2}<\mathrm{LPN}{\left(c_{t}^{i}\right)}^{*}\leq {\mathrm{st}}_{3}$, $c_{t}^{i}$ can be classified into the C2 class, if ${\mathrm{st}}_{3}<\mathrm{LPN}{\left(c_{t}^{i}\right)}^{*}$, $c_{t}^{i}$ can be classified into the C3 class. Different number of measurements can be assigned to differently classified blocks. Denote the number of measurements as ${\mathrm{SP}}_{t}^{i}$ which takes corresponding values ${\mathrm{sp}}_{0}$, ${\mathrm{sp}}_{1}$, ${\mathrm{sp}}_{2}$, ${\mathrm{sp}}_{3}$.

The values setting of ${\mathrm{st}}_{1}$, ${\mathrm{st}}_{2}$, ${\mathrm{st}}_{3}$ and ${\mathrm{sp}}_{0}$, ${\mathrm{sp}}_{1}$, ${\mathrm{sp}}_{2}$, ${\mathrm{sp}}_{3}$ is determined by the corresponding relationship between sparsity and necessary measurement number. According to the description in [23,32], these values can be determined. For ${\mathrm{st}}_{1}=5$, when $0<\mathrm{LPN}{\left(c_{t}^{i}\right)}^{*}\leq 5$, the signal can be considered as very sparse, and the corresponding number of measurements is set as ${\mathrm{sp}}_{0}=0$. For ${\mathrm{st}}_{2}=24$, when $5<\mathrm{LPN}{\left(c_{t}^{i}\right)}^{*}\leq 24$, using the mapping relationship provided in [23], the number of measurements is set as ${\mathrm{sp}}_{1}=32$. For ${\mathrm{st}}_{3}=32$, when $24<\mathrm{LPN}{\left(c_{t}^{i}\right)}^{*}\leq 32$, the number of measurements is set as ${\mathrm{sp}}_{2}=48$. When $32<\mathrm{LPN}{\left(c_{t}^{i}\right)}^{*}$, the signal is considered as non-sparse, the number of measurements is set as ${\mathrm{sp}}_{3}=64$. The sampling rate of the block equals ${\mathrm{SP}}_{t}^{i}/B^{2}$. In particular, ${\mathrm{SP}}_{t}^{i}=0$ means the block is not measured, and at the reconstruction side, a matrix with all elements of 0 is considered as the reconstruction result of this measurement.

### 3.4. Reference Block Subtraction

In video signals, there is often large redundancy between neighboring frames, so reducing the encoding codelength by exploiting the inter frame correlation is a common strategy in both traditional video encoding methods and CVS sampling methods. In this paper, using the estimated sparsity, a method to reduce the sampling rate by using the inter frame correlation in the sampling process is designed.

For an image block $x_{t}^{i}$, assume that there is a similar image block $x_{t0}^{i}$ at time t0 (1≤t0<t), we can consider $x_{t0}^{i}$ as the “reference block” of $x_{t}^{i}$. Since $x_{t0}^{i}$ is similar to $x_{t}^{i}$, by subtracting $x_{t0}^{i}$ from $x_{t}^{i}$, the signal sparsity can be effectively improved.

One of the L+1 measurement result vectors of the reference block is denoted as $\beta^{il}$. At the sampling side, since the CS domain signal $y_{t0}^{il}$ is known, thus we can set $\beta^{il}=y_{t0}^{il}$. By subtracting $\beta^{il}$ from $y_{t}^{il}$, we have $f_{t}^{il}$

(25) $$f_{t}^{il}=y_{t}^{il}-\beta^{il}=y_{t}^{il}-y_{t0}^{il}=A_{\mathrm{rw}}^{l}(x_{t}^{i}-x_{t0}^{i})=\Phi_{r}^{l}(c_{t}^{il}-c_{t0}^{il})\;$$

Similar to Equation (20), there is

(26) $$f_{t}^{i}=\sum_{l=0}^{L}f_{t}^{il}=\Phi_{r}\Psi_{w}(x_{t}^{i}-x_{t0}^{i})=\Phi_{r}(c_{t}^{i}-c_{t0}^{i})\;$$

Denote $sc_{t}^{i}=c_{t}^{i}-c_{t0}^{i}$ and $sc_{t}^{il}=c_{t}^{il}-c_{t0}^{il}$. If all elements in vector $sc_{t}^{i}$ and $sc_{t}^{il}$ are considered to be independent realizations of random variables ${\mathrm{SC}}_{t}^{i}$ and ${\mathrm{SC}}_{t}^{il}$, similar to Equations (21) and (22), the mean and variance can be estimated from

(27) $$E^{*}\left({\mathrm{SC}}_{t}^{il}\right)=\frac{\|f_{t}^{il}-\Phi_{r}^{l}d_{1}^{l}{\|}_{2}^{2}-\|f_{t}^{il}{\|}_{2}^{2}-(b_{l+1}-b_{l})n_{1}{}^{2}}{-2n_{1}(b_{l+1}-b_{l})}$$

and

(28) $$D^{*}\left({\mathrm{SC}}_{t}^{il}\right)≅\frac{\|f_{t}^{il}-\Phi_{r}^{l}d_{4}^{l}{\|}_{2}^{2}}{b_{l+1}-b_{l}}\;$$

Using the method in Section 3.3, the sparsity of $sc_{t}^{i}$, i.e., $\mathrm{LPN}{\left(sc_{t}^{i}\right)}^{*}$, can be estimated.

By comparing $\mathrm{LPN}{\left(c_{t}^{i}\right)}^{*}$ and $\mathrm{LPN}{\left(sc_{t}^{i}\right)}^{*}$, the measurement target can be decided. For a coefficient q (0<q≤1), if $\mathrm{LPN}{\left(sc_{t}^{i}\right)}^{*}<q\mathrm{LPN}{\left(c_{t}^{i}\right)}^{*}$, it can be considered that $sc_{t}^{i}$ is sparser than $c_{t}^{i}$, a shorter measurement result can be used to describe $sc_{t}^{i}$. Thus, $sc_{t}^{i}$ can be chosen as the measurement target. If $\mathrm{LPN}{\left(sc_{t}^{i}\right)}^{*}\geq q\mathrm{LPN}{\left(c_{t}^{i}\right)}^{*}$, it can be considered that the sparsity of $sc_{t}^{i}$ and $c_{t}^{i}$ is close, choosing $c_{t}^{i}$ as the measurement target and update the reference block by setting $\beta^{il}=y_{t}^{il}$ will reduce the global sampling rate.

Denote the measurement result of the current block as

(29) $$\xi_{t}^{i}=\left\{\begin{array}{c} f_{t}^{i},\;\mathrm{LPN}{\left(sc_{t}^{i}\right)}^{*}<q\mathrm{LPN}{\left(c_{t}^{i}\right)}^{*}\\ y_{t}^{i},\;\mathrm{LPN}{\left(sc_{t}^{i}\right)}^{*}\geq q\mathrm{LPN}{\left(c_{t}^{i}\right)}^{*} \end{array}\right.$$

In this section, two alternative measurement targets $c_{t}^{i}$ and $sc_{t}^{i}$ are set, by considering the sparsity of each image block, the measurement target can be selected, and the reference block is updated accordingly, so as to achieve the goal of reducing the overall video sampling rate by using the inter frame correlation. Each reference block can be updated automatically when the correlation between it and the current block becomes weak. Compared with the reference frame method, the proposed method can update the reference block more flexibly, which is also conducive to make better use of inter frame correlation to reduce the overall sampling rate. And the automatically update of reference blocks ensures a relatively small memory occupation.

### 3.5. Sampling Operations

Since CS reconstruction is not lossless and suffers from the error accumulation effect, the reconstruction quality of the reference block affects the reconstruction qualities of the corresponding blocks in the following frames, thus we expect that the reconstruction quality of the reference block could be higher than common blocks. In common blocks, the number of measurements is ${\mathrm{SP}}_{t}^{i}$, which is determined by the classification result. For reference blocks, a parameter ra (ra>1) is used to achieve a higher number of measurements. Denote the final number of measurements of blocks as

(30) $${\mathrm{SPA}}_{t}^{i}=\left\{\begin{array}{c} {\mathrm{SP}}_{t}^{i},\;\mathrm{LPN}{\left(sc_{t}^{i}\right)}^{*}<q\mathrm{LPN}{\left(c_{t}^{i}\right)}^{*}\\ {\mathrm{raSP}}_{t}^{i},\;\mathrm{LPN}{\left(sc_{t}^{i}\right)}^{*}\geq q\mathrm{LPN}{\left(c_{t}^{i}\right)}^{*} \end{array}\right.$$

where the value of ${\mathrm{SPA}}_{t}^{i}$ should not larger than $B^{2}$.

When ${\mathrm{SPA}}_{t}^{i}$ is determined, $\xi_{t}^{i}{}_{1:{\mathrm{SPA}}_{t}^{i}}$ can be transmitted to the reconstruction side, where $\xi_{t}^{i}{}_{1:{\mathrm{SPA}}_{t}^{i}}$ is a new vector composed by elements from the 1st element to the ${\mathrm{SPA}}_{t}^{i}$-th element in $\xi_{t}^{i}$. That means only a part of elements in $\xi_{t}^{i}$ is transmitted to the reconstruction side and the unnecessary part is discarded. Then the adaptive rate sampling of the current image block is completed.

Since only $\xi_{t}^{i}{}_{1:{\mathrm{SPA}}_{t}^{i}}$ is transmitted, and $\xi_{t}^{i}$ cannot be obtained by the reconstruction method, the ${\mathrm{SPA}}_{t}^{i}$ cannot be solved in the reconstruction equipment. Therefore, additional information (or the side information) including the classification result and the measurement target information should be transmitted to the reconstruction side. This increases the amount of data to be transmitted. However, for the classification result of a block, only 2 bits are needed to describe it, and the measurement target information of a block only needs 1 bit to describe. Take an image block with 64 points and each with 256 gray levels as an example, suppose that the sampling result is also quantized to 256 levels, and it has a small sampling rate, e.g., 10%. At this time, the measurement result can be described by using 64×8×0.1=51.2 bits. The additional 3 bits account for less than 6% of the total number, and with the increase of sampling rate, the proportion will further decrease. Thus, it can be considered that such additional data transmission is acceptable.

### 3.6. Reconstruction Operations

In the process of reconstruction, for a block to be reconstructed, we first take the classification result and the measurement target information from the transmitted side information, the ${\mathrm{SPA}}_{t}^{i}$ can be determined. Then we can get $\xi_{t}^{i}{}_{1:{\mathrm{SPA}}_{t}^{i}}$ from the transmitted CS sampling result. Using $\Phi_{r}{}_{1:{\mathrm{SPA}}_{t}^{i}}$ and a suitable CS reconstructed method, the $c_{t}^{i}$ or $sc_{t}^{i}$ can be reconstructed. The SPGL1 [33] method is used to reconstruct the signal here.

Assume that the reference block of current $x_{t}^{i}$ is $x_{t0}^{i}$, and a vector $b^{i}$ is used to store the reference block. In the reconstruction side, we use the reconstructed $x_{t0}^{i}{}^{*}$ to approximate $x_{t0}^{i}$, i.e., $b^{i}=x_{t0}^{i}{}^{*}$. When reconstructing $x_{t}^{i}$, if the measurement target information shows that it is $c_{t}^{i}$ to be reconstructed, the $c_{t}^{i}{}^{*}$ can be obtained from the reconstruction method. Using $\Psi_{w}^{-1}$, the reconstructed block $x_{t}^{i}{}^{*}$ can be obtained by $x_{t}^{i}{}^{*}=\Psi_{w}^{-1}c_{t}^{i}{}^{*}$. The reference block vector $b^{i}$ needs to be updated after the reconstruction is completed, with $b^{i}=x_{t}^{i}{}^{*}$. If the measurement target information shows that it is $sc_{t}^{i}$ to be reconstructed, the $sc_{t}^{i}{}^{*}$ can be obtained from the reconstruction method, and the reconstructed block can be obtained as $x_{t}^{i}{}^{*}=b^{i}+\Psi_{w}^{-1}sc_{t}^{i}{}^{*}$.

## 4. Experiments

**Figure 1:**
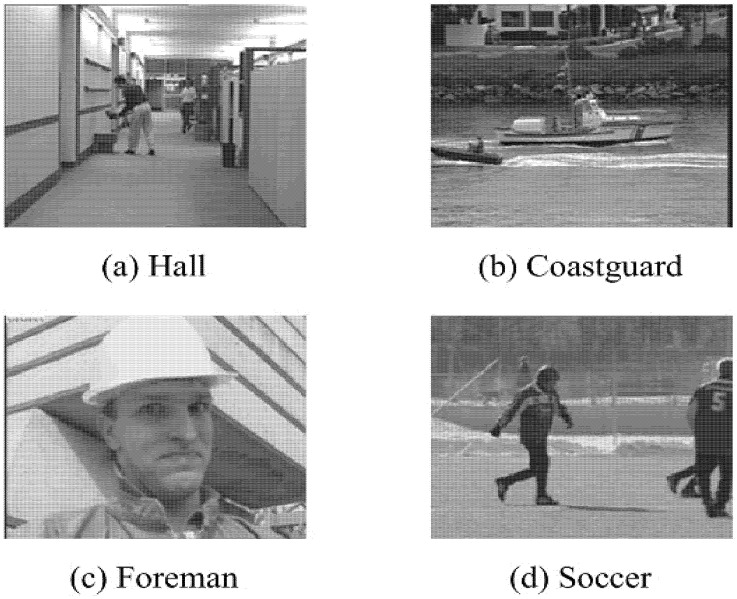
Example Frames of Test Videos.

Video sequences Hall, Coastguard, Foremen and Soccer are used to test the performance of the proposed method. Sample frames of the four test videos are shown in Figure 1.

All these videos are standard test videos, and they represent four very representative situations. Hall represents the common situation of surveillance video. Its background is constant, and the foreground is changing. Coastguard video is a typical representative of foreground object tracking. The background is changing rapidly, and the foreground is similar. Foreman video contains close-up of characters and some fast-changing scenes. The background and foreground of Soccer video are changing, and the speed of change is sometimes fast and sometimes slow. All these videos can be found from https://media.xiph.org/video/derf/ (accessed on 31 July 2021)

In this section, firstly, the parameter settings used in experiments are introduced. Next, the corresponding experiments are designed to demonstrate the image block classification ability and the sampling rate allocation ability of the proposed method, and the corresponding results are analyzed. Then, we compare the performance of the proposed method with adaptive rate CS methods proposed in recent years and analyze the results.

### 4.1. Parameter Settings

In the following experiments, Haar Wavelet Bases is used, and L=3. The Gaussian random matrix is adopted as the CS measurement matrix. 

Parameters value setting of q and ra are shown in Table 1.

The parameter q determines the update speed of the reference block. The larger the q is, the slower the reference blocks update. If the value is too large, the reference blocks will be updated very slow, the inter frame correlation will not be used effectively, and the measurements will be wasted. If the value of q is too small, the reference blocks will be updated too frequently. Because the measurement rate of the reference blocks is higher than that of the common blocks, the measurements will also be wasted.

The parameter ra determines how much higher the number of measurements of the reference block is than that of the common block. If the measurement number is too low, the quality of the whole reconstructed video will decline. If the measurement number is too high, considering the excessive measurement number contribute little to reconstruction quality, which will result in the waste of the measurement number.

The parameter values in Table 1 are obtained by our experiments, which have good effect on the four videos with different characteristics.

### 4.2. Image Block Classification Result

In the process of the adaptive rate allocation, the classification of image blocks is a key step. If the classification result fit the actual sparsity, it brings a lower sampling rate and better image reconstruction quality. Therefore, an experiment is designed to show the classification performance of the proposed method.

**Figure 2:**
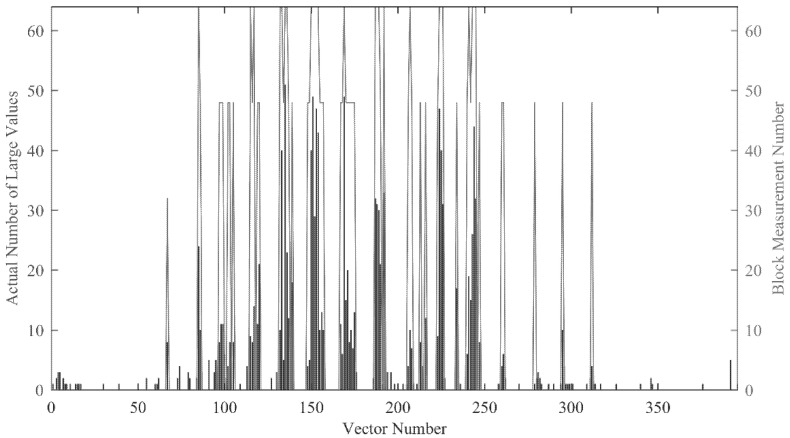
Result of measurement number allocation in a frame.

We use the classification results of all blocks in a frame to evaluate the classification performance. Take the 100th frame in the video Hall as an example. The experimental result is shown in Figure 2, the bar represents the actual number of large points, and the line represents the allocated measurement number for each block.

It should be noted that the number of measurements is theoretically larger than the number of large value points in the majority of cases. The specific correspondence is described in Section 3.3. It can be seen from Figure 2 that the allocation of measurement number basically matches the actual sparsity, and it can be adjusted according to the change of the actual sparsity. Especially for the empty blocks and non-sparse blocks, the classification results are very good. However, there are still some misclassifications in the classification results. According to our statistics, the number of misclassified blocks accounts for about 10% of the total number of blocks. Almost all misclassifications allocate higher measurement number to those blocks, which leads to the waste of measurement. However, such misclassification can ensure that the quality of the reconstructed image does not decline significantly. Generally speaking, the classification result can be considered as a quite good result.

Through the above experiment results, we can get the conclusion that the proposed method can accurately classify the image blocks only according to the known CS domain signal, and the classification results are in good agreement with the actual sparsity of the signal.

### 4.3. Measurement Number Allocation Results

Based on the classification of blocks in a frame, different number of measurements can be assigned to different blocks. The measurement number of a frame is the sum of the number of all blocks in the frame. In order to verify whether the measurement number of each frame is appropriately assigned, a relevant experiment is designed.

Using the same BCS method and the reference block subtraction strategy, the actual sparsity of the wavelet coefficients in each image block is observed, and the measurement number is determined by the actual sparsity. It is necessary to point out that when only the CS domain signal is known, the actual sparsity cannot be observed directly. The measurement number determined by the actual sparsity (named as Real measurement number) is an ideal value. By comparing the deviation between the estimated measurement number and the Real measurement number, the measurement number assignment ability of the proposed method can be evaluated.

**Figure 3:**
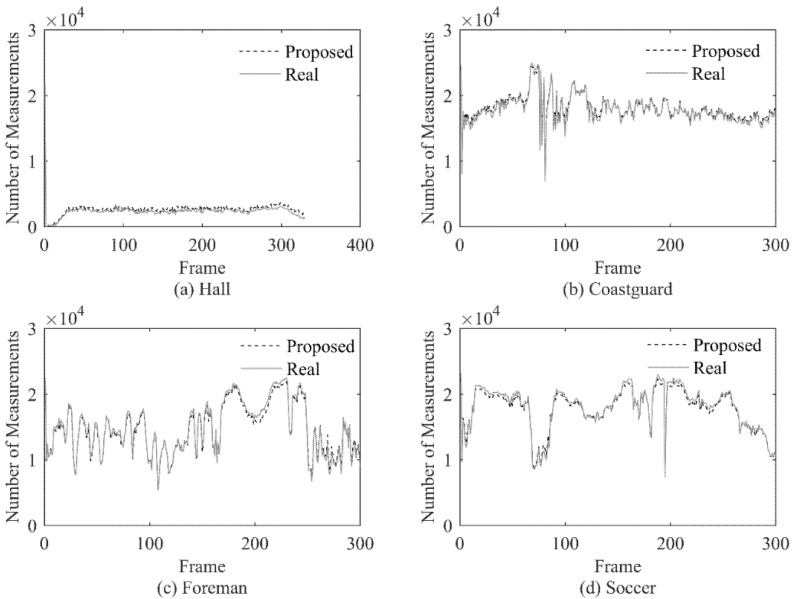
Measurement Number Comparison.

In the experiment, the real measurement number (Real) and the estimated measurement number with the proposed method (Proposed) are calculated for each frame of the 4 test videos, and the results are shown in Figure 3.

The experiment results show that the proposed method can allocate the number of measurements very well for each frame, the allocation result is very close to the ideal value, and when the actual value changes dramatically, the estimated value can also make corresponding changes according to the actual value in time. At the same time, the proposed method can well adapt to videos with different characteristics. For the four test sequences with obvious different characteristics, there is no significant gap in the performance of the proposed method.

Through the above experiment, we can get the conclusion that the proposed method can allocate an appropriate measurement number for each frame under the condition that only the CS domain signal is known.

### 4.4. Comparison of Reconstructed Image Quality

In order to evaluate the performance of the proposed method, we design another experiment to show the Peak Signal to Noise Ratio (PSNR) [34] performance of the proposed method frame by frame and compare it with the PSNR performance of several other methods. The example reconstructed images of these methods are also shown and compared.

As far as we know, there are not many adaptive rate CVS methods similar to the methods proposed in this paper. Two methods proposed in recent years are chosen for comparison, they are Compressive Domain Saliency-based Adaptive Measurement (CDSAM) [26] and Adaptive-Rate Compressive Sensing based on Fast Sparsity Estimation (ARCS-FSE) [27], respectively. The reconstructed result using Real measurement number which is mentioned in Section 4.3 is also used for comparison and named as Real.

Here, we consider the PSNR of Real as an ideal value, and hope that the PSNR of the proposed method can be close to the ideal value. In particular, it should be pointed out that the ideal PSNR value here is not necessarily the highest PSNR value. Since the goal of the adaptive method is to allocate an appropriate sampling rate, it can be considered that it is inefficient to obtain a higher PSNR with a much higher sampling rate than the actual one.

In addition, because the frame measurement number of the Real method and the proposed method are close, if the PSNRs are also close, then the intra block measurement number allocation of the proposed method can be considered as reasonable.

The CDSAM method is an adaptive rate method based on blocked CVS. It adopts a fixed frame measurement number and dynamically changes the block measurement number in the frame. Compared with the fixed rate CVS method, it has an obviously improvement in performance. According to [26], the method has the best performance compared with other adaptive rate CVS methods at that time.

ARCS-FSE is an adaptive rate CS method for surveillance videos. In the test videos used in this paper, Hall is the sequence with the characteristics of a surveillance video, so the ARCS-FSE method is applied for Hall. In other three test sequences, the ARCS-FSE method cannot be applied because of its limited applicability.

By comparing the sampling rate and the reconstructed image quality, the performance of the proposed method can be evaluated. The Real method, the proposed method and the ARCS-FSE method can adaptively determine the sampling rate for each frame, while the frame sampling rate of the CDSAM method needs to be set. Appropriate sampling rates are selected for CDSAM method so that the average PSNR of CDSAM can be close to other methods. The average sampling rates (ASR) and average PSNRs of different methods are shown in Table 2.

**Figure 4:**
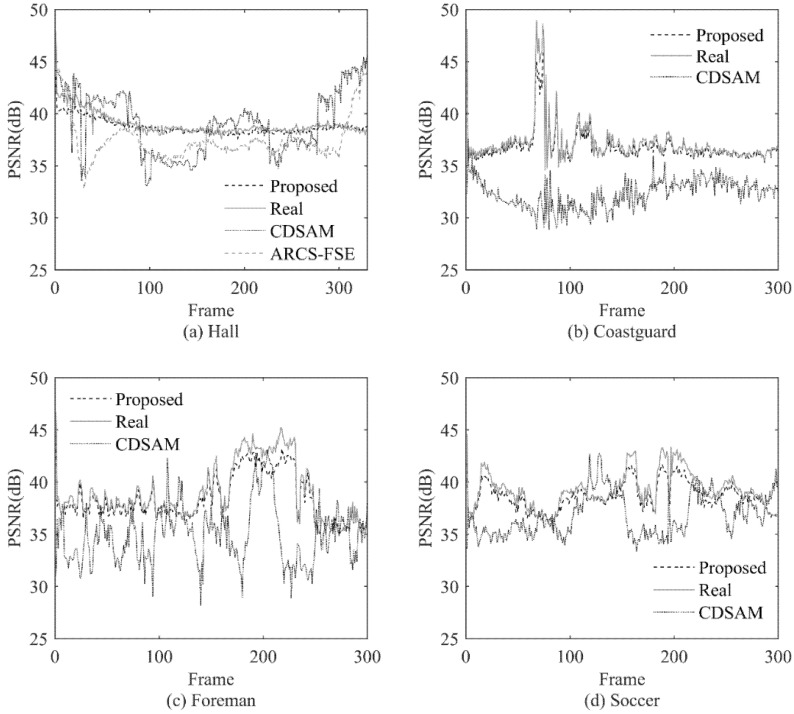
PSNR Comparison.

In order to better demonstrate the quality of reconstruction images, with the above sampling rate, PSNR of each frame for all methods are shown in Figure 4.

**Figure 5:**
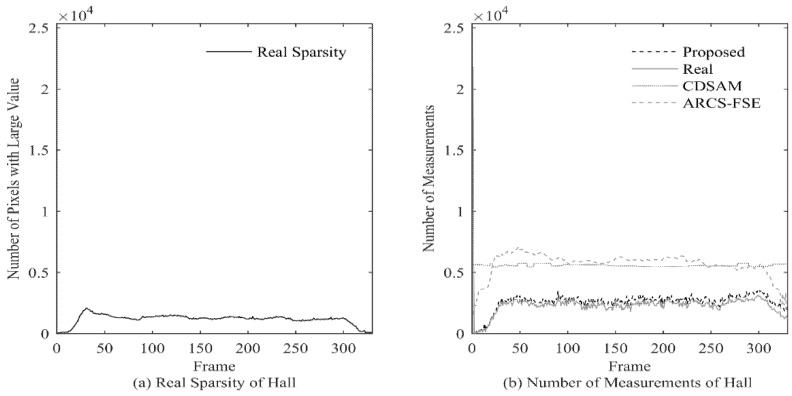
Real Sparsity and Measurement Number of Hall.

In order to further illustrate the influence of the sampling rate on PSNR, in Figure 5, we take video Hall as an example to show the actual sparsity and the measurement number of each frame.

It can be seen that the variation of proposed measurement number is consistent with the variation of actual sparsity, while CDSAM and ARCS-FSE allocate too high measurement number in some sparse regions. Combining with Figure 4a, we can see that at the end of the video, the CDSAM method achieves only a few dB of PSNR gain at a high number several times over the ideal value. At that part, because the PSNR achieved by the proposed method is relatively high, such gain is of little significance.

**Figure 6:**
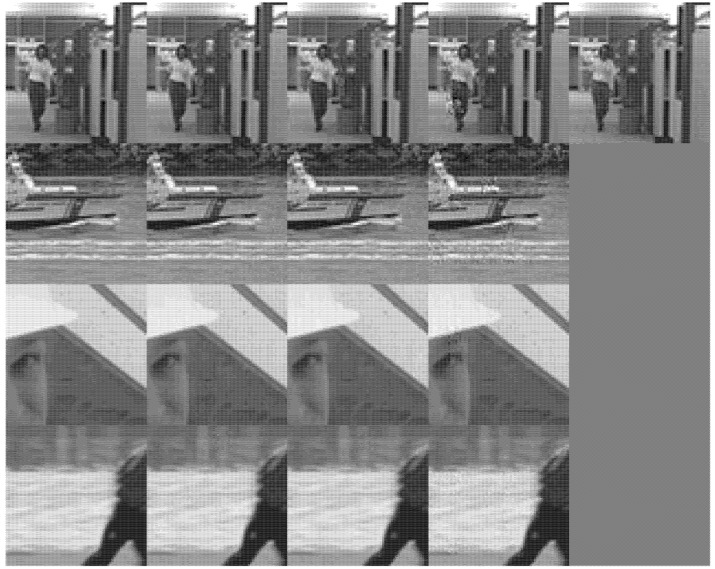
Reconstructed results of the four sequences Hall, Coastguard, Foreman and Soccer. In each line, the sample images from left to right are: the original image, the reconstructed image by Proposed, Real, CDSAM, ARCS-FSE. Since ARCS-FSE is only suitable for surveillance video, it is only used for Hall.

We also show the reconstructed frames for visual quality evaluation, a local part of the 150-th frame of each video is used as the example and shown in Figure 6.

From the above experiment results, we can see that the reconstruction quality of the proposed method is close to the Real method and has good performance. The proposed method and the Real method have close PSNR values for similar measurement number, and there is no obvious blocking effect in the visual quality of the proposed method. It shows that the measurement number allocation for each block in the proposed method is also close to the actual situation.

Compared with the CDSAM method and the ARCS-FSE method, the proposed method can obtain better reconstruction quality at a lower sampling rate. The reconstruction quality of each frame is relatively consistent. At the same time, the reconstruction quality of each block is also relatively consistent. In addition, compared with the CDSAM method and the ARCS-FSE method, the proposed method has more advantages in adapting to different videos with different characteristics.

### 4.5. Computational Complexity Discussion

First, as we discussed in Section 1, compared with the method relying on signal reconstruction, the method independent of signal reconstruction has obvious advantages in running speed. The proposed method, CDSAM and ARCS-FSE are independent of reconstruction, so they should have a great advantage in running speed compared with the methods that depend on signal reconstruction.

Secondly, the BCS scheme is used in this paper. Compared with the global measurement scheme, the measurement matrix size of BCS scheme is much smaller, which leads to less multiplication operation in the measurement process. The ARC-FSE method adopts the scheme of global measurement, which can be predicted that the proposed method should be faster in the execution speed of matrix multiplication. Compare with the CDSAM method, considering that the proposed method needs to measure each wavelet subband separately, it can be predicted that the matrix multiplication speed of proposed method will be slower than that of the CDSAM method.

Finally, as far as the sparsity estimation speed is concerned, the calculation of the proposed method is relatively simple, while the CDSAM method needs to operate on all adjacent blocks of each block in the sparsity estimation process, which makes it slower than the proposed method.

Note that the average matrix multiplication time is T1, the average sparse estimation time is T2, the average signal reconstruction time is T3, and the average sampling time of each frame is T, T=T1+T2+T3. CDSAM method, ARCS-FSE method and ARCS-CV [23] method is taken as comparison methods, where the ARCS-CV method is a representation of methods relying on signal reconstruction. Taking hall video sampling time as an example, we carried out simulation experiments on the same platform to verify the above analysis. The simulation results are shown in Table 3.

It can be seen that the simulation results are consistent with the theoretical analysis, and the proposed method has the best performance in terms of running speed in all these methods.

### 4.6. Conclusions of Experiments

It can be seen from the above experiment results that the proposed method can realize adaptive rate CVS when only the CS domain signal is known, the sampling calculation is simple. It can achieve good sampling rate adaptation and reconstructed image quality for a variety of videos with different characteristics. Compared with the existing adaptive rate CVS methods, the proposed method has obvious advantages.

## 5. Conclusions

In this paper, an adaptive rate CVS method is proposed. By only using the CS domain signal, suitable sampling rate is assigned to each image block adaptively. Experiment results show that the proposed method has better performance than the previous methods. Compared with the experimental result based on the ideal sampling rate, the proposed method can achieve the close reconstructed quality for different frames.

## Figures and Tables

**Table 1:** Parameters Setting.

Parameter	q	ra
Value	0.6	1.5

**Table 2:** Average Sampling Rate and Average PSNR (dB) of Different Methods.

	Hall ASR	Hall PSNR	Coastguard ASR	Coastguard PSNR	Foreman ASR	Foreman PSNR	Soccer ASR	Soccer PSNR
Proposed	0.1056	38.71	0.7148	36.81	0.5735	38.34	0.6996	38.63
Real	0.0923	38.98	0.7042	37.17	0.5818	38.91	0.7088	39.22
CDSAM	0.2200	39.13	0.8000	32.29	0.7000	34.89	0.8000	36.77
ARCS-FSE	0.2300	37.46	-	-	-	-	-	-

**Table 3:** Running Time of Different Methods (ms).

	$T_{1}$	$T_{2}$	$T_{3}$	T
Proposed	22.40	43.62	0	66.02
CDSAM	7.48	104.18	0	111.66
ARCS-CV	957.26	0.14	2.99 × 10^5^	3.00 × 10^5^
ARCS-FSE	788.21	0.37	0	788.58

## Data Availability

Not applicable.
